# Physical Fitness Plays a Crucial Mediator Role in Relationships Among Personal, Social, and Lifestyle Factors With Adolescents' Cognitive Performance in a Structural Equation Model. The Cogni-Action Project

**DOI:** 10.3389/fped.2021.656916

**Published:** 2021-06-14

**Authors:** Vanilson Lemes, Anelise R. Gaya, Kabir P. Sadarangani, Nicolas Aguilar-Farias, Fernando Rodriguez-Rodriguez, Clarice Maria de Lucena Martins, Camila Fochesatto, Carlos Cristi-Montero

**Affiliations:** ^1^Projeto Esporte Brasil – PROESP-Br, Escola de Educação Física, Fisioterapia e Dança, Universidade Federal do Rio Grande do Sul, Porto Alegre, Brazil; ^2^Secretaria de Estado da Educação de Santa Catarina – SED-SC, EEB Gracinda Augusta Machado, Imbituba, Brazil; ^3^Universidad Autónoma de Chile, Providencia, Chile; ^4^Escuela de Kinesiología, Facultad de Salud y Odontología, Universidad Diego Portales, Santiago, Chile; ^5^Department of Physical Education, Sports, and Recreation, Universidad de La Frontera, Temuco, Chile; ^6^UFRO Activate Research Group, Universidad de La Frontera, Temuco, Chile; ^7^IRyS Group, Physical Education School, Pontificia Universidad Católica de Valparaíso, Valparaíso, Chile; ^8^Research Centre in Physical Activity, Health, and Leisure - CIAFEL, Porto University, Porto, Portugal; ^9^Federal University of Paraíba, João Pessoa, Brazil

**Keywords:** physical fitness, body composition, cognitive performance, quality of life, vulnerability, adolescence

## Abstract

**Background:** The beneficial relationship between physical fitness and cognitive performance is affected and modulated by a wide diversity of factors that seem to be more sensitive during the development stage, particularly during early adolescence. This study aimed to examine the role of physical fitness considering the multivariate association between age, health-related quality of life (HRQOL), school vulnerability index (SVI), body mass index z-score (BMIz), physical activity, and sleep problems with the cognitive performance in boys and girls.

**Method:** Participants were 1,196 adolescents aged 10–14 years (50.7% of boys) from Chile. Three physical fitness components and eight cognitive tasks were measured. BMIz was determined using growth references by age and sex, whereas questionaries were used to assess sleep problems, physical activity, and HRQOL. SVI was established according to the score given by the Chilean Government to educational establishments. We performed a structural equation model (SEM) to test multivariate associations among study' variables by sex.

**Results:** Fitness was positively associated with boys' and girls' cognitive performance (β = 0.23 and β = 0.17; *p* = 0.001, respectively). Moreover, fitness presented a significant mediator role in the relationships between BMIz, SVI, and physical activity with cognitive performance (indirect effect). Additionally, SVI showed a negative association both direct and indirect effect in all three fitness components and all cognitive tasks, being this relationship stronger in girls than in boys.

**Conclusion:** Our findings suggest that physical fitness and all its components play a crucial mediator role in the associations between several factors associated with adolescents' cognitive performance. Thereby, educational and health strategies should prioritise improving physical fitness through physical activity. They also should address other factors such as school vulnerability, obesity, and the early gender gap in a comprehensive approach boosting cognitive performance among early adolescents.

**Trial registration:** Research Registry (ID: researchregistry5791).

## Introduction

Cognition is a complex mental process that is fundamental to acquiring knowledge, model our behaviour, and achieve personal goals (1). Several health, lifestyles, and social factors such as obesity (2), sleep problems (3), physical activity (2), physical fitness (4), social vulnerability (5), and quality of life (6) converge moderating cognitive skills throughout the entire life cycle, both positively or negatively. However, during the development stage and particularly during early adolescence, this interaction becomes essential due to their future influences (7, 8). In fact, a high cognitive performance (i.e., evaluated through diverse cognitive skills) during early stages has been associated with an improved quality of life, socioeconomic status, and mental health in adulthood (4, 9, 10).

Of all factors mentioned before, physical fitness and, particularly cardiorespiratory fitness, has been studied arduously in animal and human models because of its short- and long-term effects over several brain parameters (i.e., neuroelectric response, micro and macro brain structure, neurotrophins release), underpinning beneficial changes at the cognitive level (11–13). However, most of the literature in this research field have used regression, mediation, and moderation analyses addressing the influence of physical fitness on cognitive performance considering the diversity of lifestyles and social factors (14, 15). A more integrative and complex theoretical model such as a structural equation analysis seems to be more appropriate to improve understanding in this area due to the high simultaneous interaction among all involved factors over cognitive performance (16).

To date, various organisations and studies have declared some gaps necessary to be addressed in this critical research area. For instance, recommendations for futures studies should include diverse fitness components and cognitive tasks, explore cognitive outcomes rather than academic achievement, involve other socioeconomic status and geographical backgrounds, focus on sensible populations such as adolescents, and describe gender gaps to improve the quality and impact on this study area (17–21). Added to this, also it is relevant to contribute in this research area establishing analyses by sex (22) due to noticeable differences in pubertal hormones and brain plasticity which show great implications at a cognitive level during adolescence (23) at the physical activity level and academic achievement (24), vulnerability to poorer well-being and self-reported health (25), and other.

Therefore, this study aims to explore the role of physical fitness and its components considering the multivariate association between age, health-related quality of life (HRQOL), school vulnerability index (SVI), body mass index z-score (BMIz), physical activity, and sleep problems with the cognitive performance in a large sample of Chilean adolescents divided by sex. Hence, based on the literature, we hypothesise that all exogenous variables would be associated significantly with adolescents' cognitive performance through a fitness mediator role.

## Methods

### Study Design

This cross-sectional study was carried out from March 2017 to October 2019 as part of the Cogni-Action project (26). This project was approved by the Bioethics and Biosafety Committee of the Pontificia Universidad Católica de Valparaíso (BIOEPUCV-H103–2016) and was retrospectively registered (8/July/2020) in the Research Registry (ID: researchregistry5791). Written consents were obtained before participation from the school principal, parents, and participants. This study was prepared according to the STROBE guidelines (Strengthening the Reporting of Observational Studies in Epidemiology) for cross-sectional studies (27).

### Population, Sample Size, and Participants Selection

This study included adolescents (10–14-year-old) from the public, voucher, and private schools of the Valparaiso region, Chile. The total sample size and power calculations were based on the total pupils' enrolment in the Valparaiso region, according to the Chilean Ministry of Education in the year 2016 (universe *N* = 951,962). The sample estimation considered an alpha error of 5%, confidence interval of 99%, heterogeneity of 50%, and a 20% dropout. Thereby, 797 participants were necessary to reach a representative sample size from the second most populated region in Chile. The general inclusion criteria were girls and boys from 5th to 8th grades. Total project participants were 1,586 pupils. For this study, 1,196 pupils were included after applying the following exclusion criteria: (a) being out of the stipulated age range (*n* = 239) or (b) missed the cognitive evaluation or relevant variable (*n* = 151).

### General Measurement Procedures

All assessments (two sessions of 4 h each separated by 8 days apart) took place between 9:00 and 15:00 at schools. A complete cognitive battery was applied in the morning hours (first session) followed by anthropometric measurements and questionnaires (physical activity, sleep problems, HQOL) were evaluated. Pupils' physical fitness was measured in the second session. Trained instructors from our research team guided all evaluations, and adolescents had a brief familiarisation trial before each test.

### Cognitive Performance

The adolescents' cognitive performance was established using eight neurocognitive tasks from the NeuroCognitive Performance Test (NCPT) from Lumos Labs, Inc (28). All NCPT tasks have demonstrated adequate reliability and validity, and it is a brief, repeatable, web-based platform to measure several cognitive domains, including working memory, visuospatial memory, psychomotor speed, fluid and logical reasoning, response inhibition, numerical calculation, and selective and divided attention. The NCPT was applied in schoolrooms, in groups of 25 pupils, each one with a laptop. The entire session lasted around 1 h, which consisted of a brief explanation about the session's aim, a demonstration and practise before each test, and finally the execution. Adolescents' questions were resolved before starting each cognitive test. More details about tests are described elsewhere (26, 28).

Overall, the NCPT included: the “Trail Making A” (TMA) and “Trail Making B” (TMB) which estimate attention and processing speed, the “Forward Memory Span” (FMS) and “Reverse Memory Span” (RMS) which determine the visual short- term and working memory, respectively. The “Balance” (BAL) is a task that judges for quantitative and analogical reasoning while the “Digit Symbol Coding” (DSC) evaluates processing speed. The “Go/No-Go” (GO) task cheques for response inhibition and processing speed, and finally the “Progressive Matrices” (MAT) which assesses problem-solving and fluid reasoning. Each test was scaled following a normal inverse transformation of the percentile rank (28). These procedures provide the benefit of having scaled scores derived on the same normal distribution with a mean of 100 and a standard deviation of 15.

### Fitness Indicators

Physical fitness was evaluated by the ALPHA-fitness test battery (29), which comprises the assessment of the cardiorespiratory fitness (CRF), muscular fitness (MF), and speed-agility fitness (S-AF). This is a valid, reliable, feasible, and safe field-based fitness test battery in children and adolescents which permits evaluating and monitoring many of them simultaneously. All tests were performed in sports fields or indoor gym during mornings (second session), and adolescents wore appropriate sportswear. They practised each test previously guided by a trainer and then started when they felt secure.

### Cardiorespiratory Fitness

The 20-m shuttle run test was used to estimate the CRF. This assessment was programmed at the end of the session due to the potential fatigue induced by the test. Groups of 8–10 pupils were located at the starting line, and a sound signal indicated the run pace, which started at 8.5 km/h and increased 0.5 km/h every minute. Thus, adolescents had to run 20 m and wait on the second line until the next sound signal. To ensure a progressive increase and a correct adaptation to the test, a trainer ran beside pupils guiding the first 2 min of the test. The test ended voluntarily when the child was fatigued or unable to reach the line twice. The final stage completed (number of laps) was recorded.

### Muscular Fitness

The MF score was created based on the sum of the sex- and age-standardised handgrip/weight and standing long jump values. Handgrip was assessed twice (both hands) using a dynamometer (Jamar Plus+ Digital Hand Dynamometer, Sammons Preston, USA), which was previously adjusted to the child's hand size. This device measures between 0 and 90 kg, with a 0.1 kg precision. The child had to hold a standing position with a fully extended elbow during the evaluation. The maximum score (kg) between measures was recorded and, to create a relative measure of upper limb strength, then the score was divided by body weight (kg).

The standing long jump test was carried out to evaluate the lower limb explosive strength. A reference line was fixed on the floor, and pupils had to stand with their feet parallel behind the line. At the verbal signal, they had to jump as far as possible, always (starting and landing) with both feet at the same time. This test was performed twice (with at least 1-min rest between them), and the longest jump was recorded in centimetres (cm).

### Speed-Agility Fitness

S-AF (speed of movement, agility, and coordination) was assessed using the 4 × 10-m shuttle run test. Two lines (5 m long) separated by 10 m were fixed on the floor, and two cones were located in each line. Pupils had to run as fast as possible, taking a cloth located ~50 cm after the first line and carrying it to the next line where they had to swap for a second cloth before running to the final line. The test was performed twice, and the fastest time was recorded in seconds. Time was multiplied by −1, so a higher score indicated better performance. A z-score base on sex and age was created as a normalised S-AF score (using the same data set).

### Physical Activity

Physical activity was determined by the self-administered 7-d recall Youth Activity Profile questionnaire (YAP-SL) (30). This instrument is a simple and low-cost method to estimate moderate-to-vigorous physical activity and sedentary behaviour in children and adolescents (aged 5–17 years) through 15-item (31). These items are divided into three categories (a) physical activity in the school, (b) physical activity out of school, and (c) sedentary behaviour, which includes five periods of the day (i.e., transportation to school, activity during physical education, recess, lunch, and transportation from school). Pupils rated their responses using a 5-point Likert type scale, from 1 indicating “almost none of the free time sitting” to 5 indicating “almost all free time sitting.” Thus, a total physical activity z-score was computed for PA at school and out of school, averaging the z-scores for their components.

### Sleep Problems

Adolescents' sleep problems was evaluated with the Spanish version of the Sleep Self-Report (32). This questionnaire, which is not intended to diagnose specific sleep disorders, has 16 items that assess different areas related to sleep habits and sleep problems over a typical recent week. These items include four domains (a) sleep quality, (b) sleep anxiety, (c) bedtime refusal, and (d) sleep routines. Each item is scored with a three-point scale indicating the frequency of occurrence: usually (five to seven times a week), sometimes (two to four times a week), and rarely (never or once a week). The higher the score, the higher the sleep-related problems. A cut-off point based upon a total 16 points score was established to indicate sleep problems (1.5 standard deviations above the mean) (32).

### Fatness

Body weight was measured with a digital balance in which precision and maximum weight was of 0.1 and 150 kg, respectively (OMROM, HN-289-LA, Kyoto, Japan), whereas height was measured with a portable stadiometer (SECA, model 213, GmbH, Germany). The Body Mass Index z-score (BMIz) was then calculated using World Health Organisation 2007 growth reference for school-aged (33).

### Health-Related Quality of Life

The HQOL was determined by the self-reporting version of the KIDSCREEN-27 questionnaire (34). Overall, the questionnaire was designed to measure the health and subjective well-being of children and adolescents aged between 8 and 18 years old, and it was validated for Chilean population (35). The questionnaire includes 27 items grouped into five categories: physical well-being (five questions), psychological well-being (seven questions), relationship with parents and autonomy (seven questions), social support and peers (four questions), and school environment (four questions). More details about this questionnaire are elsewhere (34, 35).

### School Vulnerability Index

The SVI is a complex indicator which involves a great diversity of factors such as the family socioeconomic status, the educational level of parents-guardians, the student's health status, both physical and emotional well-being of children and adolescents, and the geographic location of the school. The Government of Chile has created this instrument to measure the degree of socioeconomic vulnerability of pupils who attend schools with partial or total state funding (voucher and public schools, respectively). Thereby, the SVI scores range from 0 to 100 being assigned to the private schools a score of zero (36).

### Statistical Analysis

A multivariate regression imputation for missing values was performed according to previous statistical recommendations using the maximum likelihood and robust estimator (37). The D centroid distance of the Mahalanobis test was adopted to identify multivariate normality for all variables in the present sample considering both girls and boys in this preliminary analysis. Thus, Cohen's d and independent *T*-test were used to identify possible differences between boys and girls.

**Figure 1:**
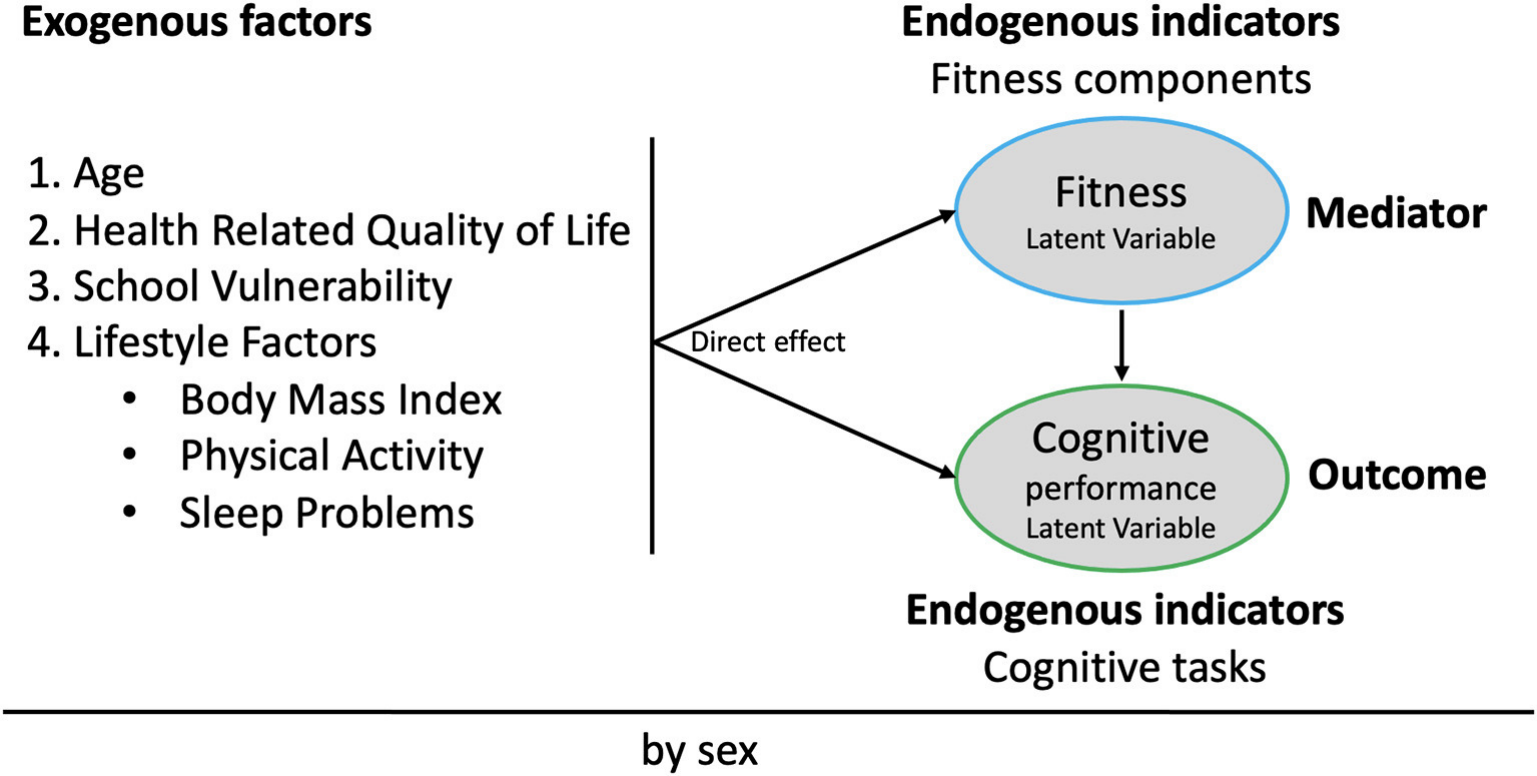
Proposed structural mediation model adapted from previous studies (38–45).

Theoretically, we have based the present study on the ecological model previously presented by Jirout et al. (38) and also on a recent systematic review (39). Overall, the general hypothesis based on the current literature is that physical fitness can be an important mediator for associations among behaviours, lifestyle, and psychosocial variables with cognitive performance. According to this, Figure 1 shows the theoretical model for multivariate associations in our structural equation model (SEM). The SEM includes the mediation path of fitness in the relationship between all exogenous factors with cognitive performance by sex (invariance model) (46). Fitness latent variable was constituted by three endogenous indicators (CRF, MF, and S-AF) whereas eight cognitive tasks constituted the cognitive performance latent variable.

Diverse parameters to verify the model sustainability were tested, and we considered goodness of fit indices as follows: standardised root means square residual (SRMR) <0.10; chi-square/degrees of freedom (CMIN/DF) <5; both the comparative fit index/incremental fit indices (CFI/IFI) and the Tucker Lewis index (TLI) approximated to 0.90, and finally the root mean square error of approximation (RMSEA) <0.08 (40, 41). All indicators are relevant to reduce potential sources of bias.

## Results

Table 1 shows descriptive adolescents' characteristics. The sample consisted of 50.7% of boys and 49.3% of girls. Significant differences were observed between boys and girls in weight, in all fitness tests (CRF, standing long jump, handgrip/weight, speed-agility), sleep problems, physical activity, and diverse cognitive tasks (TMB, Go/No-Go, Digit Symbol Coding, and Progressive Matrices). Boys showed higher values in all fitness tests and Go/No-Go compared with girls.

**Table 1:** Descriptive adolescents' characteristics.

**Variables**	**All (*****n*** **=** **1,196)**		**Boys (*****n*** **=** **606)**		**Girls (*****n*** **=** **590)**		**Sex comparison**		
	**Mean**	**SD**	**Mean**	**SD**	**Mean**	**SD**	**Cohen's d**	***t*-test**	**(*p*-value)**
Age (years)	12.21	1.05	12.18	1.03	12.25	1.06	0.07	0.961	0.337
Weight (kg)	50.28	11.88	49.35	12.00	51.24	11.70	0.16	2.75	**0.006**
Height (cm)	152.41	9.26	152.22	10.27	152.61	8.10	0.04	0.725	0.468
BMI (z-score)	1.04	1.06	1.07	1.09	1.00	1.02	0.07	1.051	0.294
HRQOL score	50.30	9.22	50.24	9.03	50.36	9.42	0.01	0.216	0.829
SVI score	56.08	35.12	57.61	34.16	54.51	36.04	0.09	1.526	0.128
Physical Activity score	0.21	4.12	0.88	4.14	−0.47	3.98	**0.33**	5.733	**0.001**
Sleep problems score	11.27	4.98	10.74	4.89	11.82	5.02	**0.22**	3.782	**0.001**
CRF (laps)	3.88	2.03	4.42	2.19	3.33	1.66	**0.56**	9.669	**0.001**
SLJ (cm)	140.59	26.55	146.77	27.62	134.25	23.81	**0.49**	8.398	**0.001**
HG/W	0.43	0.10	0.44	0.11	0.42	0.08	**0.23**	3.919	**0.001**
Speed-agility (sec)	13.04	1.31	12.75	1.34	13.33	1.22	**0.45**	7.761	**0.001**
TMA score	100.00	14.71	100.40	14.59	99.58	14.84	0.06	0.969	0.333
TMB score	100.00	14.71	98.64	14.68	101.39	14.63	0.19	3.249	**0.001**
FMS score	100.02	14.38	100.81	14.42	99.21	14.31	0.11	1.927	0.054
RMS score	99.96	14.34	100.27	14.31	99.64	14.37	0.04	0.762	0.446
GO score	100.00	14.71	101.91	14.75	98.04	14.43	**0.27**	4.59	**0.001**
BAL score	100.07	14.49	99.62	14.33	100.52	14.65	0.06	1.076	0.282
DSC score	99.99	14.67	99.15	15.03	100.86	14.26	0.12	2.016	**0.044**
MAT score	100.10	14.25	98.69	13.99	101.54	14.38	**0.20**	3.476	**0.001**

*SD, standard deviation; BMI, body mass index; HRQOL, health-related quality of life; SVI, School vulnerability; CRF, cardiorespiratory fitness; SLJ, standing long-jump; HG/W, Handgrip/weight; TMA, “Trail Making A”; TMB, “Trail Making B”; FMS, “Forward Memory Span”; RMS, “Reverse Memory Span”; BAL, “Balance”; DSC, “Digit Symbol Coding”; GO, “Go/No-Go”; MAT, “Progressive Matrices.” Z-score, standard deviation score; cm, centimetres; min, minutes; kg, kilogramme; sec, seconds. In bold, significant values with alpha <0.05 or Cohen's d > 0.20*.

### Multivariate Associations Findings

#### General Outcomes

**Figure 2:**
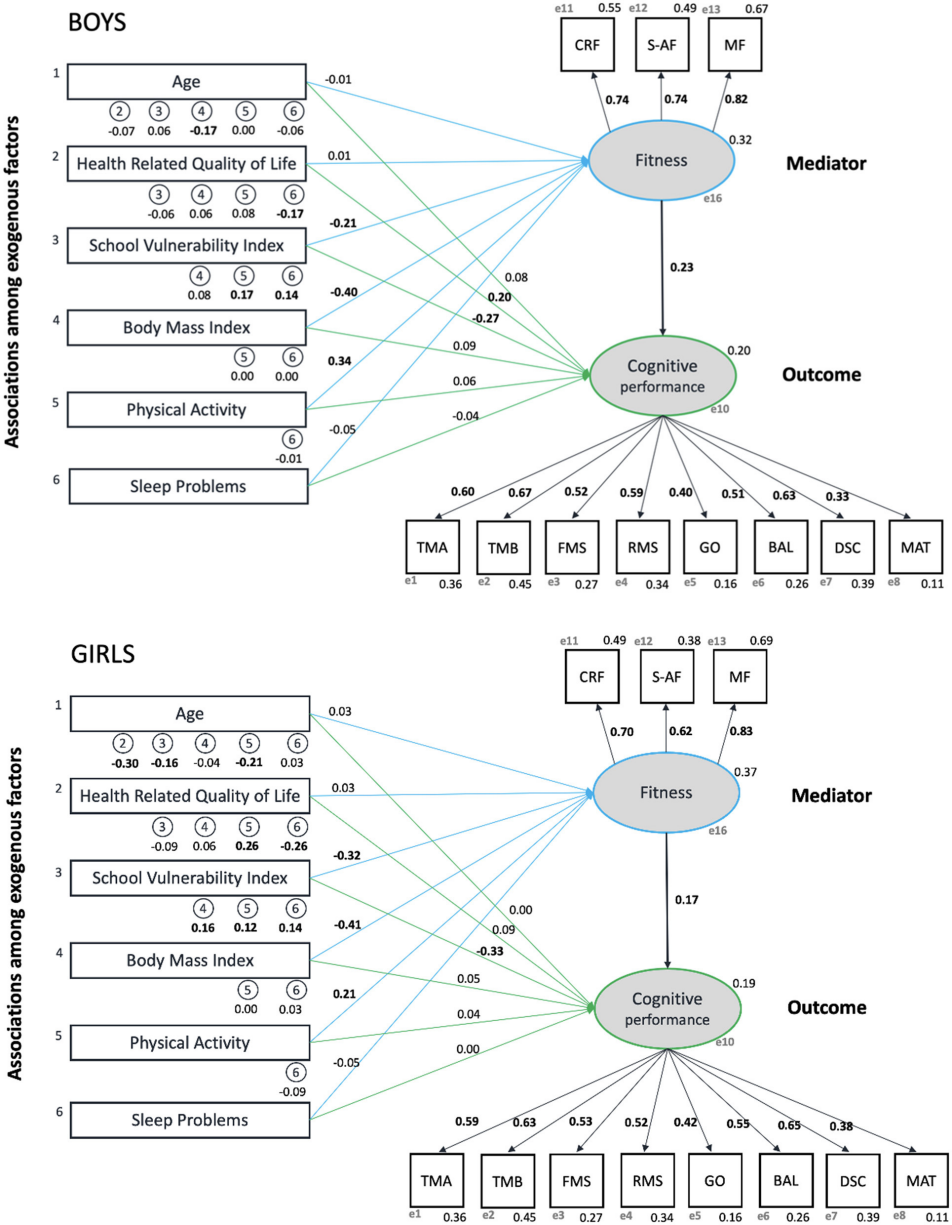
Structural equation model showing the association among different factors and the mediation role of fitness with cognitive performance in boys (superior figure) and girls (inferior figure). All values are Standardised beta coefficients. CRF, cardiorespiratory fitness; S-AF, Speed-Agility Fitness; MF, Muscular Fitness; TMA, “Trail Making A”; TMB, “Trail Making B”; FMS, “Forward Memory Span”; RMS, “Reverse Memory Span”; BAL, “Balance”; DSC, “Digit Symbol Coding”; GO, “Go/No-Go”; MAT, “Progressive Matrices.” On the left: the number in circles references the variable number. On the right: latent variables are shown as ovals, observed variables as squares, and (e) refers to endogenous indicators related to latent variables. Blue and green lines refer to a direct effect on fitness and cognitive performance, respectively. Values refer to standardised beta coefficient, and bold values are significant statically (alpha <0.05).

The SEM presented in Figure 2 was statistically adequate according to the following goodness of fit indices: SRMR = 0.0404; CMIN/DF = 2.745; CFI/IFI>0.90; TLI>0.87; RMSEA = 0.038 (0.034–0.042). Overall, the SEM showed that BMIz and physical activity were directly associated with fitness but not with cognitive performance. In contrast, SVI was directly associated with both fitness and cognitive performance. Thus, BMIz and physical activity's associations with cognitive performance would be mediated by fitness (details in Table 2). Moreover, fitness was directly related to cognitive performance in both boys and girls (β = 0.23 and β = 0.17; *p* = 0.001, respectively). Finally, concerning endogenous indicators, all three fitness components and all eight cognitive tasks contribute significantly to fitness and cognitive performance latent factors.

**Table 2:** Indirect relationships in the structural equation model for boys and girls.

**Indirect relations in boys**												
**Exogenous factors**	**fitness Endogenous indicators**			**Cognitive performance (Latent)**	**Cognitive Endogenous indicators**							
	**MF**	**S-AF**	**CRF**		**TMA**	**TMB**	**FMS**	**RMS**	**GO**	**BAL**	**DSC**	**MAT**
Age	−0.01	−0.01	−0.01	0.00	0.04	0.05	0.04	0.04	0.03	0.04	0.05	0.03
HRQOL	0.01	0.01	0.01	0.00	**0.12**	**0.13**	**0.10**	**0.12**	**0.08**	**0.10**	**0.12**	**0.07**
SVI	**−0.17**	**−0.15**	**−0.16**	**−0.05**	**−0.19**	**−0.22**	**−0.17**	**−0.19**	**−0.13**	**−0.16**	**−0.20**	**−0.11**
BMIz	**−0.33**	**−0.28**	**−0.30**	**−0.09**	0.00	0.00	0.00	0.00	0.00	0.00	0.00	0.00
Physical activity	**0.28**	**0.24**	**0.25**	**0.08**	0.01	0.01	0.01	0.01	0.01	0.01	0.01	0.01
Sleep problems	−0.04	−0.04	−0.04	−0.01	−0.03	−0.03	−0.03	−0.03	−0.02	−0.02	−0.03	−0.02
Fitness	–	–	–	**–**	**0.14**	**0.15**	**0.12**	**0.13**	**0.09**	**0.12**	**0.14**	**0.08**
**Indirect relations in girls**												
Age	0.03	0.02	0.02	0.01	0.01	0.01	0.01	0.01	0.00	0.01	0.01	0.00
HRQOL	0.03	0.02	0.02	0.01	**0.06**	**0.06**	**0.05**	**0.05**	**0.04**	**0.05**	**0.06**	**0.04**
SVI	**−0.27**	**−0.20**	**−0.23**	**−0.05**	**−0.23**	**−0.24**	**−0.20**	**−0.20**	**−0.16**	**−0.21**	**−0.25**	**−0.15**
BMIz	**−0.34**	**−0.26**	**−0.29**	**−0.07**	−0.01	−0.01	−0.01	−0.01	−0.01	−0.01	−0.01	−0.01
Physical activity	**0.18**	**0.13**	**0.15**	**0.04**	0.05	0.05	0.04	0.04	0.03	0.04	0.05	0.03
Sleep problems	−0.04	−0.03	−0.03	−0.01	−0.01	−0.01	0.00	0.00	0.00	−0.01	−0.01	0.00
Fitness	–	–	–	**–**	**0.10**	**0.10**	**0.09**	**0.09**	**0.07**	**0.09**	**0.11**	**0.06**

*All values are Standardised beta coefficients. HRQOL, Health–related Quality of Life; SVI, School Vulnerability Index; BMIz, Body Mass Index z-score; CRF, cardiorespiratory fitness; S-AF, Speed-Agility Fitness; MF, Muscular Fitness; TMA, “Trail Making A”; TMB, “Trail Making B”; FMS, “Forward Memory Span”; RMS, “Reverse Memory Span”; BAL, “Balance”; DSC, “Digit Symbol Coding”; GO, “Go/No-Go”; MAT, “Progressive Matrices.” In bold, significant values with alpha <0.05*.

#### Sex Differences

Boys and girls show similarities in their results (Figure 2). Regarding exogenous factors, girls showed more statistical associations than boys (8 in girls vs. 4 in boys). In detail by factors, age was negatively associated with BMIz in boys, whereas in girls age was associated in the same direction with HRQOL, SVI, and physical activity. HRQOL was negatively related to boys' sleep problems, while in girls, it was also related positively to physical activity. Finally, in boys and girls, SVI was linked positively to physical activity and sleep problems; however, BMIz was also associated with girls.

#### Indirect Relationships Findings

Table 2 shows indirect relationships between all exogenous factors with cognitive performance mediated by fitness. Overall, age had no indirect effect in the present model. In both, girls and boys, SVI, BMIz, and physical activity presented a significant indirect effect on cognitive performance (latent variable) through all three fitness components (CRF, MF, and S-AF); however, only SVI had an indirect effect on all eight cognitive tasks. Important to note is that BMIz and physical activity also showed a direct effect on fitness (latent variable), whilst SVI with both latent variables (fitness and cognitive performance) (Figure 2).

HRQOL had an indirect effect on all cognitive tasks; however, no indirect effect was observed with any fitness components (endogenous indicators). Remarkably, HRQOL showed a significant direct effect with latent fitness variable only in boys (Figure 2). Finally, fitness (latent variable) presented an indirect effect on all cognitive tasks.

## Discussion

### Main Study Findings

The principal result showed that the higher the fitness level, the higher the cognitive performance in 10–14-year-old adolescents, being this relationship stronger in boys than in girls. Overall, fitness and all its components (MF, S-AF, and CRF) showed a significant mediator role for the association between physical activity, BMIz, and SVI with cognitive performance. Moreover, SVI seemed to exert a negative direct and indirect effect on cognitive performance and all their tasks, also depicting a particular detrimental association with girls' fitness. Finally, our hypothesis was corroborated partially due that exogenous factors such as age, sleep problems, and HQOL were not associated with fitness, and only HQOL in boys had a direct effect over cognitive performance.

### Fitness Mediation Role on Physical Activity

Regarding the relationship between fitness and cognitive performance, several studies support the present findings. Overall, physical fitness evidences a favourable association with cognitive functions such as cognitive flexibility (47), cognitive processing (48), working memory (49), inhibitory control (50), and attention capacity (15, 51, 52). Moreover, it is possible to find some studies showing that CRF and not the physical activity is related to better cognitive performance, learning, and academic achievement (42, 43, 48, 53). We presented a new contribution in this area, suggesting an indirect relation between physical activity and cognitive performance mediated by all fitness components. Underpinning this idea, evidence has shown that the CRF influences on cognitive performance might be related to improved neural connexion, structural and functional brain outcomes, neurogenesis, and neurotrophic factor releasing (8, 39, 48). Simultaneously, MF seems to enhance neuromuscular and motor system properties (54), and S-AF could integrate CRF and MF influences. Thereby, increasing motor skills and physical activities would enhance adolescents' cognitive performance through a synergistic physiological integration among all fitness components, and not as a sole.

### Fitness Mediation Role on BMIz

BMIz showed a negative direct effect on fitness and an indirect effect on cognitive performance. In children and adolescents, obesity has been linked to worse mental health, academic achievement, and cognitive functions (55), which is more prevalent in low-middle income countries (56). This is a major issue in Chile as 51.7% of children and adolescents are overweight or obese, one of the highest prevalence worldwide (57).

High adipose tissue storage, mainly at the visceral level, promotes a low-grade systemic inflammation related to lower cognitive performance in children (58, 59). Cytokines and hormones (i.e., CRP, IL-6, Leptin) play a relevant role in brain functioning; hence paediatric obesity would affect brain structure and function (58, 60). However, it has been shown that physical fitness counteracts the effect of obesity meditating on various physiopathological processes, for instance, inducing an anti-inflammatory mechanism at the organic level (61), and also attenuating the genetic predisposition to obesity in children and adolescents (62, 63). A study in children and adolescents with overweight and obesity showed that CRF and S-AF were associated with several cortical and subcortical brain structures, even after controlling for BMI and other covariates (62). Hence, although it is recognised that an adequate balance between fatness and fitness is crucial to promote a better academic achievement and cognitive performance (55, 64); based on the literature and our findings, we could establish that fitness plays a pivot role between fatness and cognitive performance. Indeed, the current study showed the non-direct association between BMIz and cognitive performance.

### Fitness Mediation Role on SVI

SVI was the strongest exogenous factor in our model. SVI is a close proxy of poverty and socioeconomic status, two moderators associated with a detrimental influence over cognitive functions (5, 7). Our results showed that SVI was associated positively with sleep problems and physical activity (boys and girls). To date, it is well-known that poor quality sleep (e.g., sleep duration, not meeting the international recommendations according to age) is associated with reduced volumes in some regions of the brain (hippocampus, prefrontal cortex, and cingulate gyrus) (3), which in turn, is related to unfavourable children and adolescents cognitive profile (65, 66). A study in adolescent athletes showed that those who participated in higher-intensity sports presented better sleep quality (67). Moreover, the only research, including physical activity, sleep quality, and cognitive performance in one model (in adults) found that sleep quality mediates the association between physical activity and cognitive performance (68).

On the other hand, SVI was negatively related to BMIz in girls (exogenous associations) and SVI presented a direct and indirect effect on fitness and cognitive performance. Hence, school vulnerability aggravates the imbalance between fatness and fitness, which reduced cognitive function both in the short and long term (4, 40, 55). This critical vicious circle among vulnerability, fitness, and fatness must be controlled early, reducing the probability of lower socioeconomic status, mental health, and quality of life in adulthood (69).

Despite this discouraging scenario, it is possible to speculate, based on our findings, that the positive association between SVI and physical activity could ameliorate the negative influence on cognitive performance through a chain of simultaneous events related to improved fitness, sleep quality, and reduced fat mass (67, 70, 71). In this sense, (72) through a mediation analysis found that physical fitness mediates the adverse relationship between BMI and cognitive performance, and this findings seems to be independent of parental socioeconomic status (72). Thereby, physical activity programs in children and adolescents with unfavourable social backgrounds and, mainly, in girls, should be a comprehensive strategy to improve their executive function and cognitive performance (73).

### Sex Differences

One of the most relevant differences between sexes was the direct and indirect effect of HRQOL on cognitive performance and all eight tasks in boys. In girls, only an indirect effect was observed. On the one hand, the literature on children and adolescents from Latin-American countries using HRQOL is scarce (74); in this sense, this study contributes to bridging the gap at the geographical level. A study in Spanish schoolchildren between 12 and 17 years old showed several differences across HRQOL's domains between boys and girls (75). On the other hand, HRQOL is a factor related to diverse domains such as physical and psychological well-being, relationship with parents and autonomy, social support and peers, and school environment (34). These sort of affective, emotional, and social domains can modulate brain development and in turn, adolescent's executive functions (6). Regrettably, physical fitness showed no mediation over HRQOL in both boys and girls. Thus, improving quality of life is an enormous challenge in this field of knowledge, even more in countries with high social inequities.

Finally, there are three other findings which are relevant to consider reinforcing our analysis by sex. First, only in girls, a significant and negative association between physical activity level and age was observed; second, the negative relationship between SVI with both fitness and cognitive performance was stronger in girls than in boys, and third, SVI was associated positively with BMIz, but particularly in girls. Therefore, these outcomes highlight an early stage sex gap (41, 69) and demonstrate the relevance and priority for implementing programs to promote healthy lifestyles (increase physical activity, fitness, and prevent obesity), emphasising opportunities to girls (75, 76).

### The Structural Equation Model

Unlike other structural equations which have included a cognitive outcome in their models (1, 77–82), to our knowledge, this is probably the first model that considered multiple relationships among age, HRQOL, SVI, BMIz, physical activity, sleep-problems, several fitness components, and a diversity of cognitive tasks in a large adolescents sample from a Latin-American country.

Overall, most of these studies using SEM have explored the mediator role of executive functions between physical activity or fitness, and academic achievements, and have been conducted in samples between 80 and 601 children or adolescents aged 6–12 years-old (1, 78, 80, 81). Two studies have used a more comprehensive model, including factors such as socio-demographics, family context, lifestyles, gender, and parental education (77, 82). However, the study by Padulo et al. (82) has focused on academic achievement and the study by Syväoja et al. (77) on an executive function (only reaction time).

Moreover, most SEM studies addressing this research area are from developed countries such as Italy (82), France (78), New Zealand (79), The Netherlands (1, 81), Switzerland (80), and Finland (77). Thereby, one of the most challenging tasks in our study is to contrast our results with evidence that emerged from a more favourable social and educational contexts (70). The socioeconomic factors are crucial for understanding the interaction among the adolescents' social context and their health and educational background (14). Therefore, strength in our model is to have included SVI as a powerful socioeconomic and social marker, which played a leading role in our findings.

Finally, all these differentiating characteristics complicate, to some extent, possible comparisons between our findings with other SEMs because it is difficult to find studies using similar variables, population age, evaluation methods, cultural identity, and others. However, our findings provide a starting point for researchers and decision-makers in developing countries to consider the social context when designing schools' educational strategies or public health policies for enhancing adolescents' cognitive skills. Thus, a practical message for educators and school principals is to change their gaze toward novel strategies that can improve the cognitive performance of schoolchildren and, in turn, academic performance, such as opportunities for increasing school physical activity, improving adolescents' physical fitness, and enhancing body composition. Some strategies have been reviewed elsewhere (83). This suggestion should focus on adolescents belonging to the highest vulnerability groups and girls to reduce the gender gap.

### Strengths and Limitations

This study included a relatively large adolescent population and provided evidence from a usually underrepresented region in this research area (17, 70). Besides, it involved a wide diversity of tasks related to cognitive performance that denote a consistent theoretical basis, considering the weights of intervenient variables such as school vulnerability, sex, and age. Moreover, we measured different fitness components, capturing dimensions that have not been explored previously. These features allowed us to develop a complex SEM to understand better how factors are interconnected. However, our findings should be considered with certain parsimony and caution due to the cross-sectional nature, which does not establish causality. Add to this, some variables could be affected by self-report bias (i.e., physical activity, sleep problems, and HRQOL) which could be resolved using objective methods in some of them in the near future. Also, we were unable to measure other factors that have been associated with cognitive performance (e.g., diet, motor skills, self-esteem, blood markers of systemic inflammation, brain-derived factors, and others); nonetheless, the inclusion of more variables in SEM could be detrimental for fit indices. Finally, longitudinal and experimental studies are necessary to corroborate our results.

## Conclusions

This study concludes that physical fitness and all its components showed a significant mediating role in the relationship between physical activity, SVI, BMIz, and cognitive performance. SVI seems to be the foremost independent factor affecting directly and indirectly adolescents' cognitive performance. Thereby, public policies promoting physical activity and reducing fatness while considering and controlling SVI are decisive actions for ensuring enhanced pupils' cognitive profile mediated by all physical fitness components. Simultaneously, strategies should also consider reducing inequities between boys and girls.

## Data Availability Statement

The raw data supporting the conclusions of this article will be made available by the authors, without undue reservation.

## Ethics Statement

The studies involving human participants were reviewed and approved by Bioethics and Biosafety Committee of the Pontificia Universidad Católica de Valparaíso (BIOEPUCV-H103–2016). Written informed consent to participate in this study was provided by the participants' legal guardian/next of kin.

## Author Contributions

CC-M contributed to the design of the project. CC-M and VL conceptualised the design of the study, analysed the data, and wrote the concept version of the manuscript. AG, KS, NA-F, FR-R, CM, and CF critically reviewed the manuscript and edited the paper. All authors have given final approval of the manuscript and agreed to be accountable for the accuracy and integrity of any part of the work.

## Conflict of Interest

The authors declare that the research was conducted in the absence of any commercial or financial relationships that could be construed as a potential conflict of interest.
